# Identification of histological carotid plaque vulnerability by CT angiography using perivascular adipose tissue radiomics signature

**DOI:** 10.1186/s13244-025-02134-y

**Published:** 2026-01-05

**Authors:** Keqiang Shu, Junye Chen, Kang Li, Xiaoyuan Fan, Liangrui Zhou, Chaonan Wang, Leyin Xu, Yanan Liu, Yuyao Feng, Deqiang Kong, Xiaojie Fan, Bo Jiang, Jiang Shao, Zhichao Lai, Bao Liu

**Affiliations:** 1 Department of Vascular Surgery, Peking Union Medical College Hospital, Chinese Academy of Medical Sciences & Peking Union Medical College, Beijing, China; 2 Department of Radiology, Peking Union Medical College Hospital, Chinese Academy of Medical Sciences & Peking Union Medical College, Beijing, China; 3 Department of Pathology, Peking Union Medical College Hospital, Chinese Academy of Medical Sciences & Peking Union Medical College, Beijing, China; 4 Department of Hemangioma and Vascular Malformation, Plastic Surgery Hospital, Chinese Academy of Medical Sciences & Peking Union Medical College, Beijing, China

**Keywords:** Atherosclerosis, Computed tomography angiography, Radiomics, Carotid stenosis, Machine learning

## Abstract

**Objectives:**

This study aims to develop a radiomics model based on carotid perivascular adipose tissue (PVAT) from CT angiography to identify histologically confirmed vulnerable plaques in patients with carotid artery stenosis (CAS).

**Materials and methods:**

In this prospective cohort study, we enrolled patients with CAS scheduled for carotid endarterectomy between 2014 and 2023. Histological plaque assessment served as the reference standard for vulnerability. We developed three models: the PVAT attenuation model, the conventional plaque feature model, and the PVAT radiomics model using features extracted from segmented CT images and machine learning. Model performance was evaluated using the area under the receiver operating characteristic curve (AUC), calibration, and decision curve analysis across training, validation, and independent testing from three different scanners. Shapley Additive exPlanations (SHAP), a tool that quantifies the contribution of each feature to the model’s predictions, was used to enhance model interpretability.

**Results:**

We included 122 patients (mean age 66.45 years, 81.97% male, 63.11% vulnerable). In the training, validation, and testing sets, the PVAT radiomics model predicts an AUC of vulnerability of 0.945, 0.819, and 0.817, respectively, while the plaque score model showed an AUC of 0.688, 0.799, and 0.497, and the PVAT attenuation model showed an AUC of 0.667, 0.708, and 0.493, respectively. The PVAT radiomics model outperforms the PVAT attenuation model (*p* = 0.01) and plaque score models (*p* = 0.03) in the test set. SHAP analysis highlighted significant predictors such as *logarithm_firstorder_RootMeanSquared*.

**Conclusions:**

The PVAT radiomics model is a promising non-invasive tool for identifying vulnerable carotid plaques, offering superior diagnostic efficacy and generalizability across different imaging equipment.

**Critical relevance statement:**

The carotid PVAT radiomics identified histologically vulnerable plaques before surgery through an interpretable and generalizable machine-learning model, beneficial for risk stratification and surgical decision-making.

**Key Points:**

- Noninvasive and effective identification of histological carotid vulnerable plaques is challenging.
- The PVAT radiomics outperforms conventional imaging biomarkers in identifying vulnerable plaques.
- The PVAT radiomic model is generalizable across scanners and interpretable, assisting clinical decision-making.

**Graphical Abstract:**

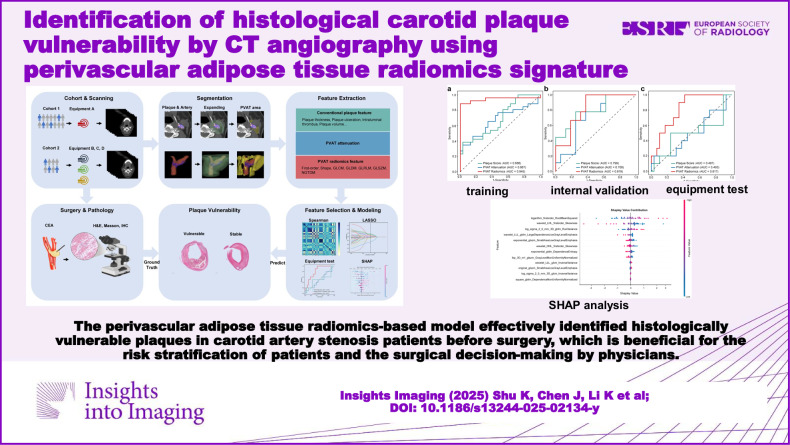

## Introduction

Stroke remains a leading cause of death and disability worldwide [1], with carotid atherosclerosis being a major contributing factor. Randomized controlled trials have demonstrated that carotid endarterectomy (CEA) reduces stroke risk in selected patients with carotid artery stenosis (CAS) [2–4]. Since vulnerable plaques significantly increase stroke risk, their identification is critical for surgical decision-making. Histologically, vulnerable plaques are characterized by features such as a large lipid-rich necrotic core, thin fibrous cap, ulceration, thrombosis, intraplaque hemorrhage, and extensive inflammatory cell infiltration [5]. However, histological confirmation is only possible postoperatively.

Computed tomography angiography (CTA) has emerged as a frontline imaging modality for assessing carotid atherosclerosis, enabling evaluation of both stenosis severity and plaque characteristics [6]. Certain qualitative and quantitative plaque features provide additional insights into plaque vulnerability [7–10], yet conventional CT parameters lack sufficient sensitivity, practicality, and generalizability for clinical use. Thus, novel imaging biomarkers are urgently needed to improve the identification of vulnerable CAS patients.

Recent studies suggest that vascular inflammation suppresses lipid accumulation in perivascular adipose tissue (PVAT) [11]. This phenomenon can be detected on routine CTA as increased CT attenuation [11], which demonstrated predictive value for plaque progression and cardiovascular mortality in coronary artery disease [12, 13]. Compared with coronary PVAT, carotid PVAT is more prone to irregular anatomical morphology and white adipose tissue phenotype [14]. Several studies have reported that carotid PVAT density is associated with clinical symptoms and imaging-based plaque risk characteristics [15–18]. However, these studies lack the histological assessment of plaque specimens as the reference standard. Moreover, the single feature of density is far from sufficient to characterize the heterogeneity of carotid PVAT, resulting in limited diagnostic value.

Radiomics offers a promising solution by enabling high-throughput extraction of quantitative imaging features through computational analysis [19]. This approach involves mining thousands of quantitative features from medical images and applying machine learning (ML) to uncover clinically relevant patterns [19]. We hypothesized that PVAT radiomics features could correlate with and help identify vulnerable carotid plaques.

In this prospective cohort study, we aimed to develop a CT-based carotid PVAT radiomics ML model to identify histologically confirmed vulnerable plaques, outperforming conventional plaque features and PVAT attenuation. Furthermore, we validated the model’s generalizability across different scanners and enhanced its interpretability using Shapley Additive Explanations (SHAP) analysis.

## Materials and methods

### Study protocol

**Fig. 1:**
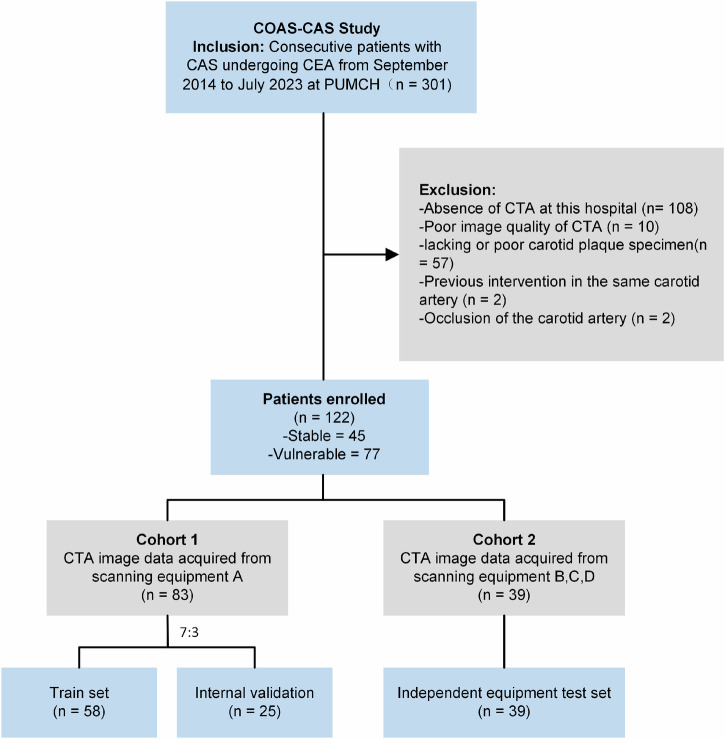
The flow chart for patient screening. COAS-CAS study, the optimization strategy of carotid artery stenosis; PUMCH, Peking Union Medical College Hospital; CTA, CT angiography

This study complies with the Declaration of Helsinki and was approved by the Institutional Ethics Review Board of Peking Union Medical College Hospital (PUMCH) (no. JS-2966). All study participants provided written informed consent. The Cohort of Optimized Assessment Strategy of Carotid Artery Stenosis (COAS-CAS) (Clinical trial: NCT05629000) is a prospective study to investigate the optimized imaging or other means of assessing patients with CAS. Patients with CAS undergoing CEA were consecutively included from September 2014 to July 2023 at PUMCH from the COAS-CAS study. Exclusion criteria were the absence of CTA imaging, poor image quality, lacking or inadequate carotid plaque specimens, previous intervention in the same carotid artery, or carotid artery occlusion. Cohort 1 consisted of patients scanned by equipment A, while cohort 2 consisted of patients scanned by equipment B, C, and D. Cohort 1 was randomly split into a training set and an internal validation set with a 7:3 ratio, while cohort 2 was used as an independent equipment testing set. Figure 1 describes the patient selection.

### Patient characteristics

We obtained demographic, clinical information (e.g., comorbidities, symptoms, cerebrovascular status, and degree of carotid stenosis), patient CTA data, and CEA plaque specimens. Symptomatic patients were defined as those experiencing neurological symptoms such as transient ischemic attacks or cerebral infarction within six months before surgery, as confirmed by neurologists or vascular surgeons (Table 1).

**Table 1:** Demographics and clinical characteristics in all patients

Variables	Total (*n* = 122)	Vulnerable (*n* = 77)	Stable (*n* = 45)	*p*
Age	66.45 ± 7.74	67.05 ± 7.47	65.42 ± 8.16	0.26
BMI	24.73 ± 2.69	24.97 ± 2.60	24.31 ± 2.82	0.19
Sex				0.36
Male	100 (81.97)	65 (84.42)	35 (77.78)	
Female	22 (18.03)	12 (15.58)	10 (22.22)	
Hypertension	84 (68.85)	55 (71.43)	29 (64.44)	0.42
Diabetes	44 (36.07)	29 (37.66)	15 (33.33)	0.63
Hyperlipemia	48 (39.34)	32 (41.56)	16 (35.56)	0.51
CAD	32 (26.23)	20 (25.97)	12 (26.67)	0.93
MI	6 (4.92)	5 (6.49)	1 (2.22)	0.54
Smoking	79 (64.75)	53 (68.83)	26 (57.78)	0.22
TC	3.62 ± 0.84	3.60 ± 0.82	3.64 ± 0.88	0.82
HDL	0.99 ± 0.23	0.98 ± 0.22	1.01 ± 0.23	0.51
LDL	2.00 ± 0.67	1.99 ± 0.67	2.03 ± 0.68	0.71
TG	1.09 (0.89, 1.73)	1.16 (0.91, 1.81)	0.99 (0.85, 1.52)	0.28
Symptom				0.10
Asymptomatic	84 (68.85)	49 (63.64)	35 (77.78)	
Symptomatic	38 (31.15)	28 (36.36)	10 (22.22)	
NASCET degree	0.73 ± 0.10	0.72 ± 0.10	0.74 ± 0.11	0.44
NASCET category				0.80
50–69%	47 (38.52)	29 (37.66)	18 (40.00)	
70–99%	75 (61.48)	48 (62.34)	27 (60.00)	
ECST degree	0.79 ± 0.09	0.80 ± 0.08	0.78 ± 0.10	0.24

Categorical variables are presented as a number (%). Continuous variables are presented as mean ± standard deviation or median (interquartile range)

*BMI* body mass index, *CAD* coronary heart disease, *MI* myocardial infarction, *TC* total cholesterol, *HDL* high-density lipoprotein, *LDL* low-density lipoprotein, *TG* triglycerides, *NASCET* the North American Symptomatic Carotid Endarterectomy Trial, *ECST* the European Carotid Surgery Trial

### CTA scanning parameters and protocols

All carotid CTA examinations were performed using the institutional routine protocol. Patients were randomly selected to use different scanning equipment. Scanning parameters of the four equipment A, B, C, and D, are summarized in Table 2.

**Table 2:** CT scanning parameters

	Equipment A	Equipment B	Equipment C	Equipment D
Manufacturer	SIEMENS	GE	Philips	Canan
Model name	SOMATOM Definition Flash	Discovery CT750 HD	IQon Spectral CT	Aquilion ONE
Distribution				
Train	58	0	0	0
Internal validation	25	0	0	0
External test	0	8	6	25
CT parameter				
Tube voltage (kVp)	90	120	120	100
Tube current (mA)	171–495	188–600	103–370	328–440
Gantry revolution time	0.25	0.5	0.5	0.5
Helical pitch	0.7	0.984375	1.015	0.806
Acquisition mode	Spiral	Spiral	Spiral	Spiral
Section thickness	1	0.625	1	0.5
Kernel	Qr40d\3	STANDARD	B	FC43
Matrix	512 × 512	512 × 512	512 × 512	512 × 512
CTDIvol median (mGy)	4.85	32.96	9.4	15.8
DLP median (Gy × cm)	194.3	1454.71	403.3	745.3
Contrast media injection parameters				
Contrast media	Iopamidol (370 mgI/mL, Bracco)			
Volume (mL)	80			
Injection rate (mL/s)	5.0			
Time of scanning	Automatic bolus starting 4 s after threshold (100 HU) was reached in the ascending aorta or aortic arch			
Saline flush after contrast media	50 mL at 5 mL/s			

*CTDIvol* volume computed tomography dose index, *DLP* dose length product

### Surgical specimen and histological analysis

The acquisition, preprocessing, and staining of carotid plaque specimens and the assessment of plaque Vulnerability are detailed in the Supplementary Material.

### Assessment of conventional plaque features in CT

Plaque thickness (including total plaque, soft plaque, and calcified plaque thickness), rim sign, spotty calcification, plaque ulceration, intraluminal thrombus, plaque burden, plaque length, total plaque density (HU), total plaque volume (mm³), calcified plaque volume (mm³), and calcification proportion were measured. The definition and measurement of features are presented in the Supplementary Material.

### Segmentation, image preprocessing, and radiomic feature extraction

**Fig. 2:**
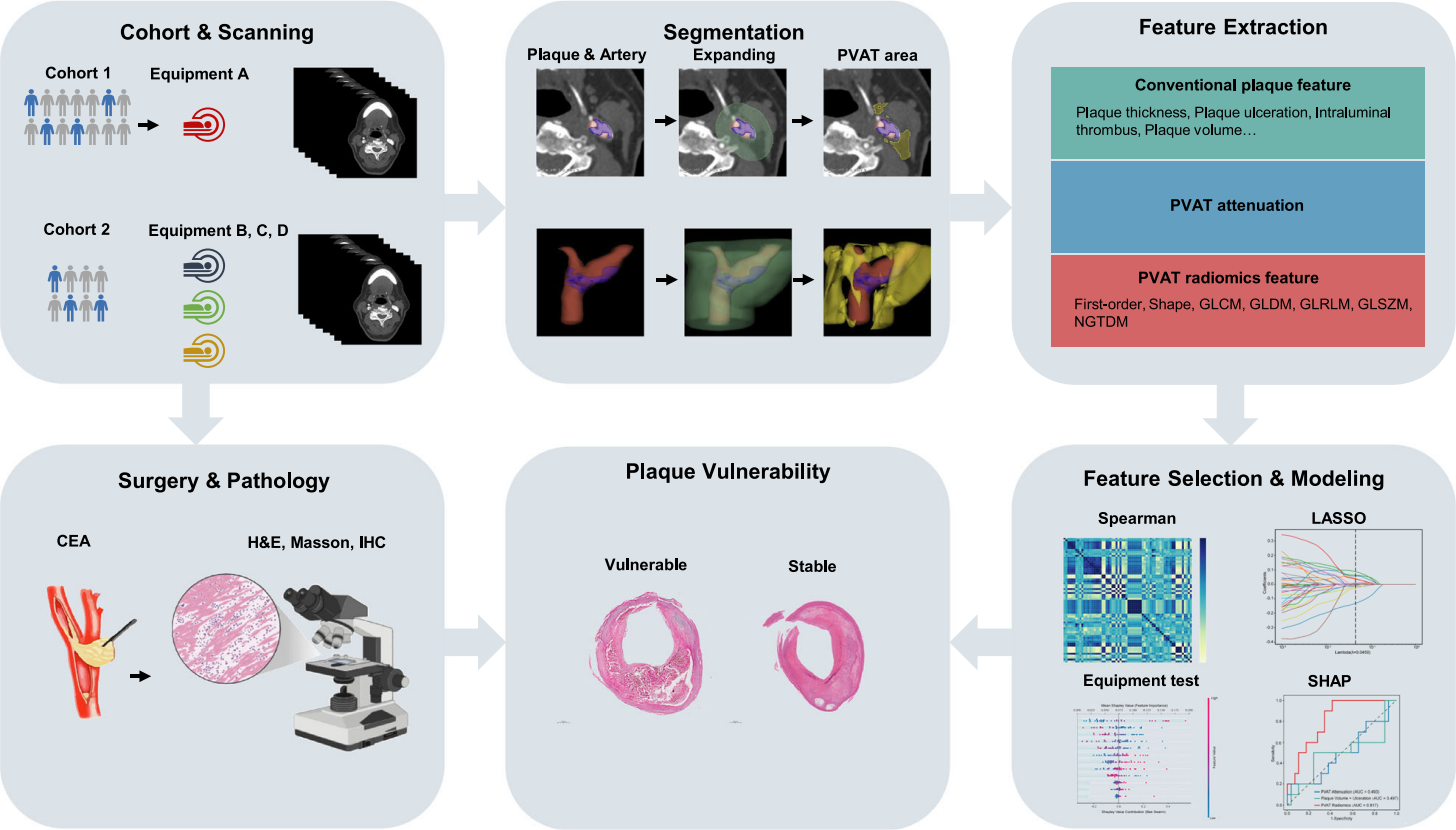
The workflow of conventional feature and radiomics analysis. PVAT, perivascular adipose tissue; CEA, carotid endarterectomy; GLCM, gray-level co-occurrence matrix, GLDM, gray-level dependence matrix, GLRLM, gray-level run length matrix, GLSZM, gray-level size zone matrix, NGTDM, neighborhood gray-tone difference matrix; H&E, hematoxylin-eosin staining; IHC, immunohistochemistry; LASSO, least absolute shrinkage and selection operator; SHAP, Shapley additive explanations

The workflow of radiomics analysis is shown in Fig. 2. Regions of interest (ROIs) were manually delineated using the open-source software 3D Slicer (version 5.0.3). ROIs for carotid plaques and artery lumen were labeled in multiple layers in short-axis, while PVAT surrounding the carotid artery was semi-automatically segmented. The PVAT segmentation covered an area within 2 cm above and below the carotid bifurcation, extending radially outward by 9 mm [20], with CT values between −30 and −190 HU [12]. Subcutaneous fat tissue and other irrelevant regions were manually excluded. Physician A (K.S., with 4 years of experience in vascular imaging) labeled the PVAT and plaques with masks. After that, Physician B (X.F., with 8 years of experience in vascular imaging, respectively) double-checked the preliminary labeling results and relabeled cases. When disagreements occurred, Physician C (B.L., with 30 years of experience in vascular imaging) made the final decision. All physicians were blinded to the clinical information and histological results of the patients.

Before feature extraction, preprocessing was applied to the images and labels. The anatomical orientation of the images and labels was initially standardized to align with the RAS (right-anterior-superior) axis codes. Resampling all images and labels to an isotropic voxel size of 1 × 1 × 1 mm³ using bilinear interpolation for the images and nearest neighbor interpolation for the labels. To reduce variability caused by differences in acquisition methods and devices, images were normalized by *z*-score.

Radiomic features were extracted from the segmented images using the open-source library “*PyRadiomics*” (version 3.8.8) implemented in Python. A total of 6 categories and 1834 handcrafted features were extracted, including first-order features, shape features, the Gray-Level Co-Occurrence Matrix, Gray-Level Dependence Matrix, Gray-Level Run Length Matrix, Gray-Level Size Zone Matrix, and Neighborhood Gray-Tone Difference Matrix, as shown in Fig. S1.

To assess inter-observer consistency, images from 50 randomly selected patients were re-segmented by Physician D (Y.L., with 4 years of experience in vascular imaging), and radiomic features were extracted again. The consistency of radiomic features extracted by the two observers was evaluated using the intraclass correlation coefficient (ICC).

### Radiomic feature selection and ML model construction

Radiomic features in the training set were standardized using the *z*-score method, with the same mean and standard deviation values applied to standardize the validation and test sets. Features with an ICC > 0.75 were considered highly stable and reproducible and were retained for further analysis. The radiomic feature selection process involved several steps: first, features with statistically significant differences (*p* < 0.05) between vulnerable and stable plaque groups were identified using a *t*-test. Second, Pearson correlation analysis was used to eliminate redundant features with a correlation coefficient greater than 0.9, followed by further feature reduction using a greedy recursive elimination strategy that systematically removed the most redundant features in each iteration. Third, the least absolute shrinkage and selection operator (LASSO) regression method, based on 10-fold cross-validation, was applied for further feature refinement, with the penalty coefficient (λ) chosen based on the minimum binomial deviation plus one standard deviation.

To build the ML model, the final set of selected radiomic features was used to construct predictive models with logistic regression (LR), support vector machine (SVM), and random forest (RF) algorithms. The most stable ML algorithm was employed to construct the radiomic model, and the radiomic score (Rad-score) was calculated using the formula: Rad-score = ln(*p*/(1 − *p*)), where *p* represents the probability of vulnerable plaque predicted by the radiomic model for each patient.

### Statistics and model interpretability

Statistical analyses were performed using SPSS (version 26.0), the Scikit-learn package in Python, and R software (version 4.0.0). Categorical variables were described as percentages and frequencies, while continuous variables were presented as mean ± standard deviation (SD) or median with interquartile range (IQR). Normal continuous variables were compared using Student’s *t*-test, skewed continuous variables were analyzed using the Mann–Whitney *U*–test, and categorical variables were assessed using the Chi-square test. Univariate and Multivariate LR analysis were used to identify independent risk factors for plaque vulnerability among conventional plaque features (*p* < 0.05), which were then used to construct an LR model. The discriminative performance of the models was evaluated using receiver operating characteristic (ROC) curves and the area under the curve (AUC). Model calibration was assessed with calibration curves, and clinical utility was analyzed using decision curve analysis (DCA). Diagnostic metrics, including accuracy, sensitivity, specificity, positive predictive value (PPV), and negative predictive value (NPV), were calculated for each model. Comparisons of AUCs between ROC curves were performed using the DeLong test. All statistical tests were two-sided, and a *p*-value < 0.05 was considered statistically significant. SHAP analysis was conducted to interpret the contributions of individual radiomics features in the PVAT radiomic model.

## Results

### Patient characteristics

**Fig. 3:**
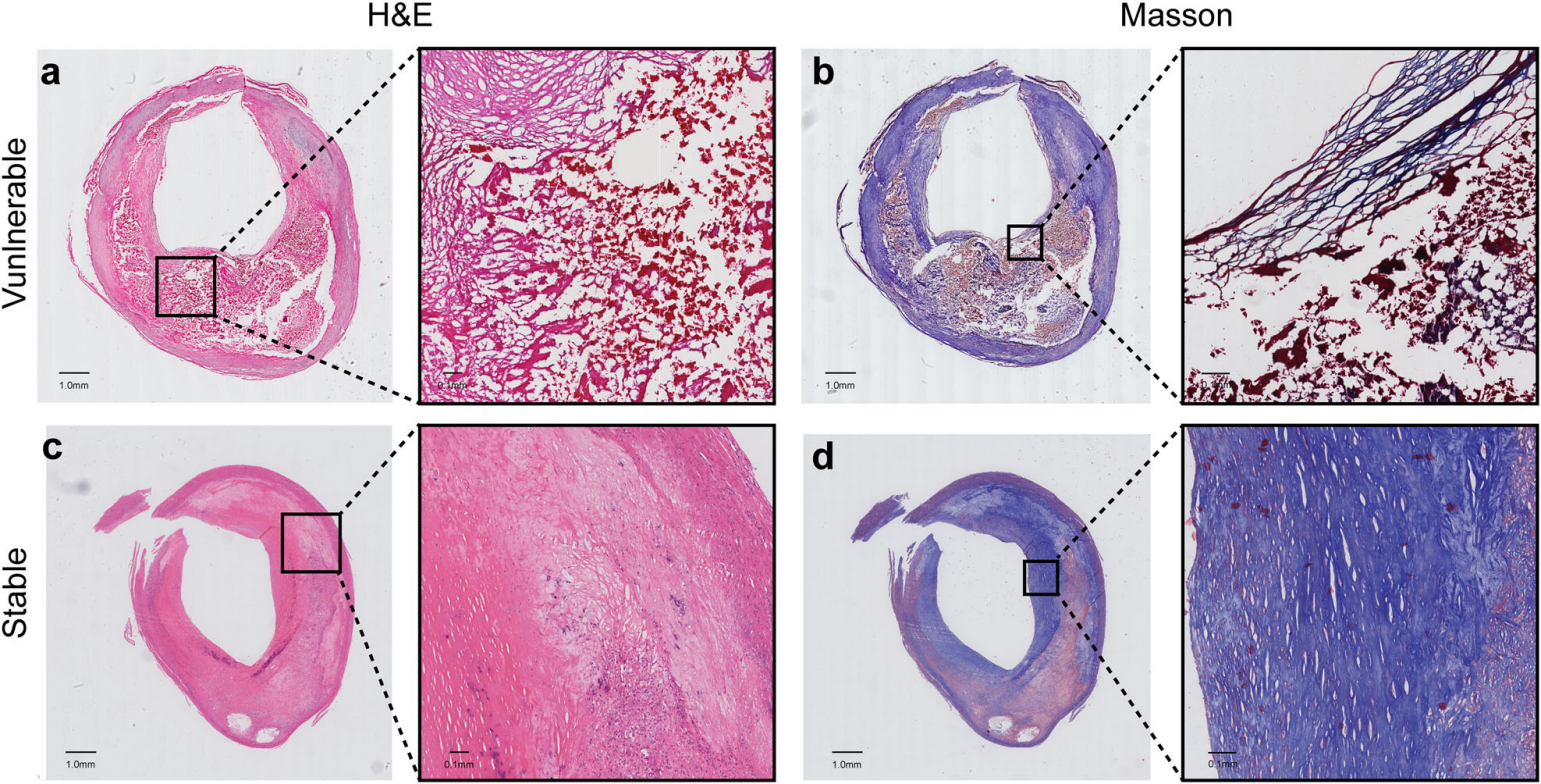
Representative histological images of the vulnerable and stable carotid plaques. H&E (**a**) and Masson (**b**) staining of a vulnerable plaque with a thin fiber cap, large lipid-rich necrotic core, and intraplaque hemorrhage. H&E (**c**) and Masson (**d**) staining of a stable plaque with a thick fiber cap and small pools of extracellular lipid. H&E, hematoxylin-eosin

We ultimately enrolled 122 patients, 83 in cohort 1 and 39 in cohort 2. The clinical characteristics of the patients (such as demographics, comorbidity, blood lipid, symptoms, and stenosis severity) are summarized in Tables 1 and S1. Among all patients, 77 had vulnerable plaques, while 45 had stable plaques. Examples of histological images of vulnerable and stable plaques are shown in Fig. 3. The Mean age of all patients was 66.45 years (age range, 48–82 years), and 22 of them were female (18.03%). The mean NASCET stenosis degree of all carotid arteries included was 73%. Among the arteries, 47 (38.52%) presented with moderate stenosis (50–69%), 75 (61.48%) with severe stenosis (70–99%), and 38 (31.15%) were symptomatic. No significant differences in demographic or clinical characteristics were observed between vulnerable and stable plaque groups (Table 1) or across the training, internal validation, and external test sets (Table S1).

Four CT scanners were utilized, namely Siemens SOMATOM Definition Flash (Equipment A), GE Discovery CT750 HD (Equipment B), Philips IQon Spectral CT (Equipment C), and Canon Aquilion ONE (Equipment D). For sample distribution, Equipment A included 58 training and 25 internal validation cases, while Equipment B, C, and D had 8, 6, and 25 external test cases, respectively. CT parameters show general differences between scanning equipment, such as tube voltage, current, section thickness, and so on (Table 2).

### Conventional plaque features and PVAT attenuation

A total of 15 conventional plaque imaging features were analyzed. Across all patients, soft plaque thickness (*p* = 0.046), plaque ulceration (*p* = 0.03), and total plaque volume (*p* = 0.03) were significantly associated with plaque vulnerability (Table S2). Additionally, PVAT attenuation was significantly higher in patients with vulnerable plaques compared to those with stable plaques (*p* = 0.005) (Table S2). To identify predictive features for plaque vulnerability, univariate and multivariate LR analyses were conducted in Cohort 1 (Table 3). Plaque ulceration (univariate, *p* = 0.03; multivariable, *p* = 0.03), total plaque volume (univariate, *p* = 0.01; multivariable, *p* = 0.01), and PVAT attenuation (univariate, *p* = 0.003; multivariable, *p* = 0.01) were confirmed as independent predictors of plaque vulnerability. Based on these findings, plaque ulceration and total plaque volume were used to construct a Plaque Score Model via LR, while PVAT attenuation was incorporated into a separate PVAT Attenuation Model. Furthermore, no differences in plaque features and PVAT attenuation were observed among different data sets (Table S1) or scanners (Table S3).

**Table 3:** Univariable and multivariable analyses of conventional plaque features and PVAT attenuation in Cohort 1

Variables	Vulnerable (*n* = 48)	Stable (*n* = 35)	Univariable^a^		Multivariable^b^	
			*p*	OR (95% CI)	*p*	OR (95% CI)
Conventional plaque features						
Total plaque thickness (mm)	5.05 ± 1.45	4.52 ± 1.52	0.11	0.78 (0.57–1.06)		
Soft plaque thickness (mm)	4.00 ± 1.68	3.41 ± 1.55	0.11	0.80 (0.60–1.05)		
Calcified plaque thickness (mm)	1.81 (1.41, 2.31)	1.68 (0.00, 2.19)	0.52	0.88 (0.60–1.30)		
Calcified plaque	15 (31.25)	9 (25.71)	0.58	0.76 (0.29–2.02)		
Rim sign	15 (31.25)	11 (31.43)	0.99	1.01 (0.39–2.58)		
Spotty calcification	9 (18.75)	8 (22.86)	0.65	1.28 (0.44–3.75)		
Plaque ulceration	11 (22.92)	1 (2.86)	0.03^*^	0.10 (0.01–0.81)	0.03^*^	0.09 (0.01–0.80)
Intraluminal thrombus	1 (2.08)	4 (11.43)	0.11	6.06 (0.65–6.84)		
Total plaque density (HU)	168.88 (95.51, 262.80)	162.38 (102.23, 287.54)	0.62	1.00 (1.00–1.00)		
Total plaque volume (mm^3^)	705.87 (519.83, 1036.61)	535.46 (244.24, 844.18)	0.01^*^	0.99 (0.99–0.99)	0.01^*^	0.99 (0.99–0.99)
Calcified plaque volume (mm^3^)	213.79 (101.37, 399.79)	186.32 (42.59, 418.23)	0.37	1.00 (1.00–1.00)		
Calcification proportion	0.33 (0.17, 0.56)	0.33 (0.18, 0.55)	0.74	1.33 (0.25–7.20)		
Plaque burden	0.88 (0.83, 0.93)	0.89 (0.82, 0.93)	0.11	0.05 (0.00–2.02)		
Plaque length (mm)	19.36 (15.55, 23.18)	19.28 (13.47, 22.09)	0.18	0.96 (0.91–1.02)		
Remodeling index	2.29 ± 0.96	2.30 ± 1.16	0.94	1.02 (0.67–1.55)		
PVAT feature						
PVAT attenuation (HU)	−68.64 ± 6.31	−73.76 ± 7.92	0.003^*^	0.90 (0.84–0.97)	0.01^*^	0.91 (0.84–0.98)

*PVAT* perivascular adipose tissue, *OR* odds ratio, *CI* confidence interval

Categorical variables are presented as a number (%). Continuous variables are presented as mean ± standard deviation or median (interquartile range)

^a^ Univariable means univariable LR

^b^ Multivariable means variables < 0.05 in the univariate LR were included for multivariate LR

^*^p < 0.05, indicating that the difference was statistically significant

### Radiomic feature selection

A total of six categories comprising 1834 handcrafted features were extracted, including 360 first-order features, 14 shape features, and the remaining texture features (Fig. S1). After statistical filtering, 64 features were retained (*p* < 0.05), with their corresponding *p*-values illustrated in Fig. S2. Following Spearman correlation analysis (Fig. S3), the highly correlated features were removed (Spearman’s correlation coefficient > 0.9), and 36 features were retained. Ultimately, LASSO LR identified 12 features with nonzero coefficients, which were used to construct the Rad-score (Fig. S4a). The coefficients and the mean standard error from the 10-fold cross-validation process are shown in Fig. S4b, c.

### Model performance

**Fig. 4:**
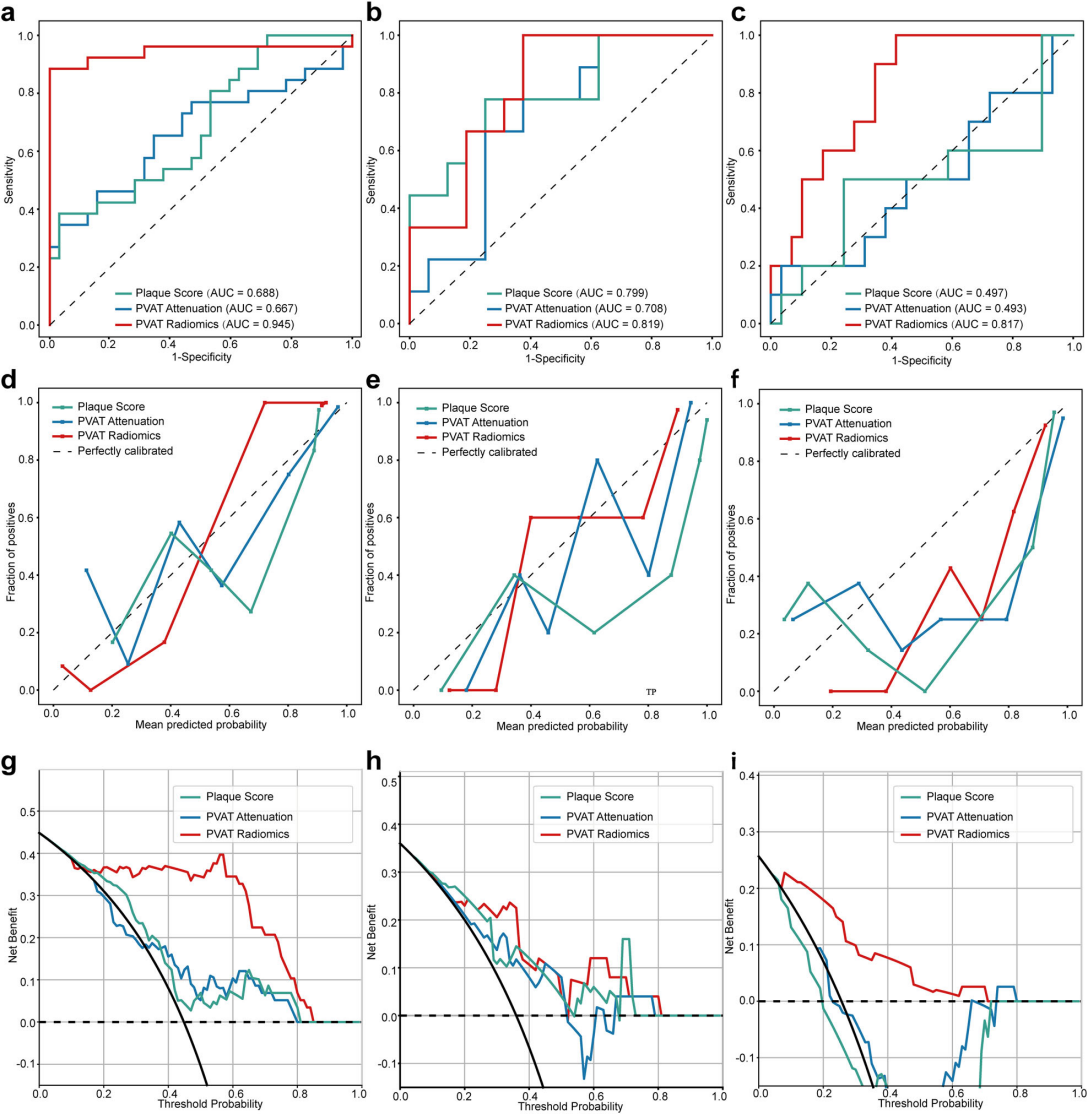
ROC curve, calibration curve, and clinical decision curve. ROC curve of the training set (**a**), internal validation set (**b**), and independent equipment test set (**c**); calibration curve of the training set (**d**), internal validation set (**e**), and independent equipment test set (**f**). Clinical decision curve of the training set (**g**), internal validation set (**h**), and independent equipment test set (**i**). ROC, receiver operating characteristic

The ROC curves for the PVAT attenuation, plaque score, and PVAT radiomic model were demonstrated to evaluate their discriminative performance in the training, validation, and test sets (Fig. 4a–c). Model performance was quantified using metrics such as accuracy, AUC, sensitivity, specificity, PPV, and NPV (Table 4). The PVAT radiomic model using the SVM algorithm demonstrated excellent discrimination, achieving an AUC of 0.945 (95% CI: 0.866–1.000) in the training set, 0.819 (95% CI: 0.654–0.985) in the validation set, and 0.817 (95% CI: 0.682–0.952) in the test set. SVM-based radiomic model outperforms those based on LR (AUC = 0.892, 0.729, 0.676, respectively) and RF (AUC = 0.839, 0.795, 0.672, respectively) algorithms, thus SVM was chosen as the final algorithm for the radiomics model (Fig. S5). In contrast, the PVAT attenuation and plaque score models exhibited moderate performance in the validation set (AUC = 0.708 and 0.799, respectively) but significantly declined in the test set (AUC = 0.493 and 0.497, respectively). In DeLong’s test, although no statistically significant differences were observed among the models in the internal validation set (*p* > 0.05), the PVAT radiomic model outperformed the PVAT attenuation and plaque score models both in the training set (Radiomics vs plaque score, *p* = 0.001; Radiomics vs PVAT attenuation, *p* = 0.002) and test set (Radiomics vs plaque score, *p* = 0.03; Radiomics vs PVAT attenuation, *p* = 0.01). No significant differences were found between the PVAT attenuation and plaque score models in any dataset (Table 5).

**Table 4:** Performance of the models in identifying vulnerable plaques

Models	AUC	95% CI	Accuracy	Specificity	Sensitivity	NPV	PPV
Training							
PVAT radiomic	0.945	0.866–1.000	0.931	0.846	1.000	1.000	0.889
PVAT attenuation	0.667	0.518–0.816	0.672	0.308	0.969	0.889	0.633
Plaque score	0.688	0.549–0.826	0.690	0.346	0.969	0.900	0.646
Internal validation							
PVAT radiomic	0.819	0.654–0.985	0.720	0.889	0.625	0.571	0.909
PVAT attenuation	0.708	0.499–0.918	0.680	0.556	0.750	0.556	0.750
Plaque score	0.799	0.605–0.992	0.720	0.667	0.750	0.600	0.800
Equipment independent test							
PVAT radiomic	0.817	0.682–0.952	0.667	0.900	0.586	0.429	0.944
PVAT attenuation	0.493	0.265–0.722	0.744	0.100	0.966	0.500	0.757
Plaque score	0.497	0.252–0.742	0.667	0.400	0.759	0.364	0.786

Categorical variables are presented as a number (%). Continuous variables are presented as mean ± standard deviation or median (interquartile range)

*PVAT* perivascular adipose tissue, *AUC* area under the curve, *CI* confidence interval, *PPV* positive predictive value, *NPV* negative predictive value

**Table 5:** Delong test among the plaque score model, PVAT attenuation model, and PVAT radiomic model

Data set	PVAT radiomic vs plaque score	PVAT radiomic vs PVAT attenuation	Plaque score vs PVAT attenuation
Train	0.001^*^	0.002^*^	0.84
Internal validation	0.86	0.41	0.51
Equipment independent test	0.03^*^	0.01^*^	0.99

*PVAT* perivascular adipose tissue

^*^p < 0.05, indicating that the difference was statistically significant

Calibration curves demonstrated the fit of the three models across the training, validation, and test sets (see Fig. 4d–f). DCA further demonstrated that the PVAT radiomic model provided greater net benefit across probability thresholds, particularly in the test set (see Fig. 4g–i).

### Model interpretability

**Fig. 5:**
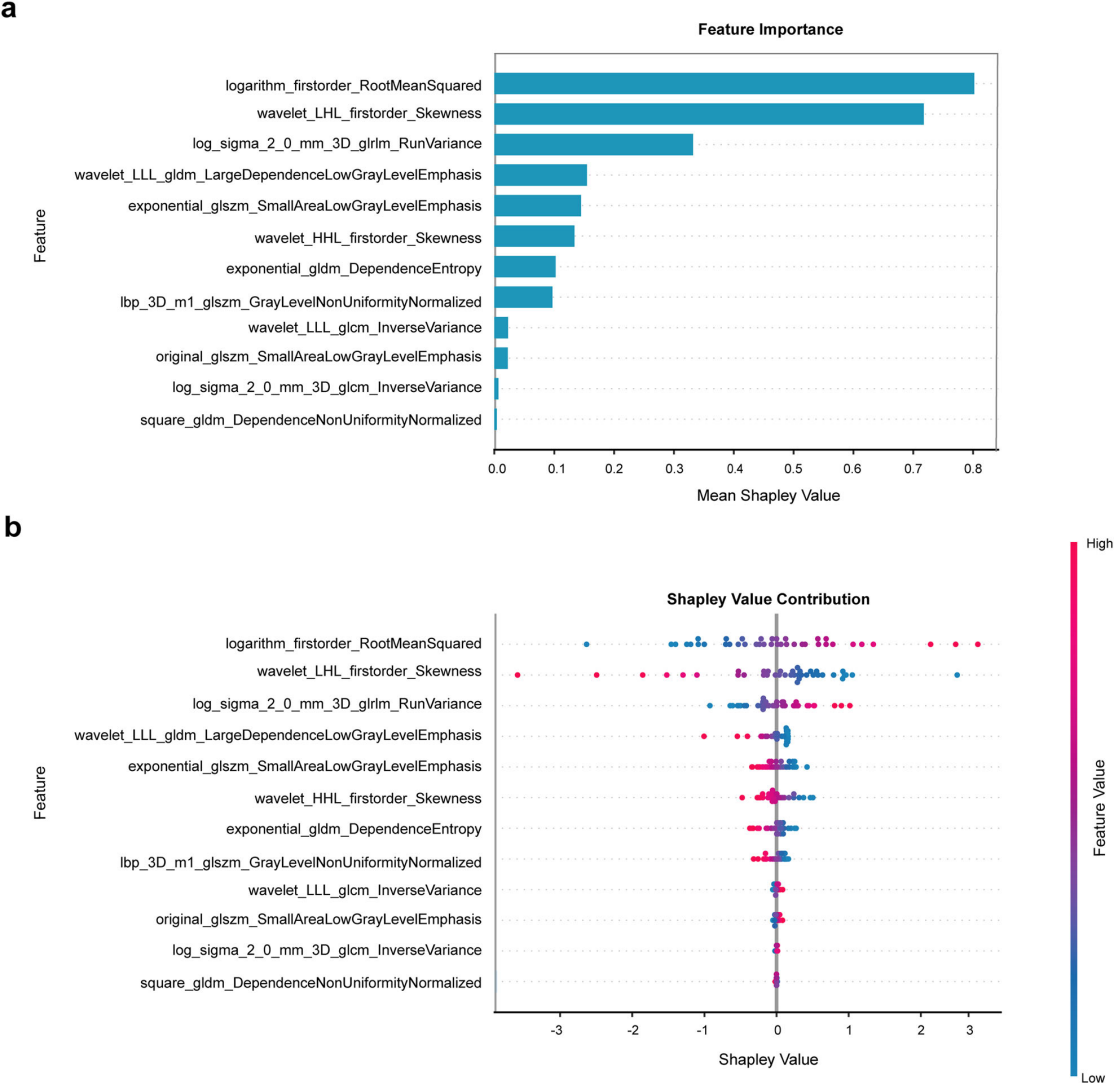
The SHAP analysis of the PVAT radiomics model in the test set. The importance of 12 radiomic features on the model’s predictive performance (**a**). The SHAP summary plot displays the SHAP values distribution of each radiomic feature (**b**). SHAP, Shapley additive explanations

SHAP analysis provided quantitative insights into the carotid PVAT radiomic ML model. SHAP values for each feature were visualized through variance importance and summary plots (Fig. 5a, b). Among these features, *logarithm_firstorder_RootMeanSquared*, *wavelet_LHL_firstorder_Skewness*, and *Log_sigma_2_0_mm_3D_glrlm_RunVariance* had the highest mean Shapley values. The other 9 features also contributed to the model.

## Discussion

In this study, we developed a predictive model based on the radiomic signature of carotid PVAT derived from CT angiography, designed to identify patients with histologically vulnerable plaque. The model demonstrated superior performance compared to plaque score and PVAT attenuation values. Additionally, the model showed robust generalizability when tested on an independent dataset from different screen equipment. Shapley analysis demonstrates critical radiomic features reflecting tissue heterogeneity and addressing black-box issues in ML.

Previous studies have shown that plaque ulceration, the most critical image sign of luminal surface irregularities, is associated with cerebrovascular events [4, 21]. Similarly, carotid plaque volume correlates with vulnerability, progression, and future cerebrovascular events [22–24]. However, conventional plaque features lack direct inflammatory indicators. Emerging evidence indicates that PVAT composition reflects vascular inflammation, with CTA-derived biomarkers (e.g., Fat Attenuation Index) capturing inflammation-mediated adipocyte size and lipid content changes [4, 11]. Other studies have also shown that carotid PVAT density is associated with some features of vulnerable plaques, like symptoms and intraplaque hemorrhage detected by Magnetic Resonance Imaging [15–17]. In our study, PVAT attenuation and plaque scores showed comparable performance in identifying vulnerable plaques. However, PVAT radiomics outperformed conventional plaque features, suggesting high-dimensional PVAT analysis may better characterize plaque vulnerability. This may be attributed to the calcification-related CT amplification effect, which diminishes the precision of voxel values and spatial distributions in conventional plaque analysis [25]. These findings align with coronary studies demonstrating PVAT radiomics’ superior predictive value for acute coronary syndrome [26].

Histological assessment of plaque vulnerability (the reference standard) demonstrates our model’s clinical value for surgical decision-making. While recent studies of carotid PVAT/plaque radiomics have focused only on symptomatic status [18, 27–29]—readily available but clinically limited—we targeted histologically confirmed vulnerability, such as large necrotic core and thin-cap fibroatheroma [30], using intraoperative specimens to develop preoperative PVAT radiomic predictors.

Scanner variability significantly impacts imaging model generalizability, yet this factor has been largely overlooked in carotid imaging studies. Reiazi R et al highlighted significant dependency of radiomic features on scanner parameters [31], and Chen Y et al reported low reproducibility of radiomic features between single-energy and dual-energy CT [32]. These findings highlight the need for multi-scanner validation. Our evaluation across three different CT systems showed: (1) significant AUC declines for PVAT attenuation and plaque score models, likely due to sensitivity to parameters like tube voltage, current, and slice thickness variations [33, 34]; and (2) maintained performance of the PVAT radiomic model, demonstrating superior robustness across imaging scanner platforms.

To improve interpretability, we used SHAP values to explain the PVAT radiomics ML model. Of the 12 most predictive features identified, three reflected grayscale distribution and nine represented texture characteristics, suggesting radiomics can detect certain histological changes missed by mean PVAT attenuation. SHAP analysis identified three dominant discriminative features in patients with vulnerable plaque: (1) elevated logarithm_firstorder_RootMeanSquared (suggesting PVAT heterogeneity and inflammation probably), (2) reduced wavelet_LHL_firstorder_Skewness, and (3) increased log_sigma_2_0_mm_3D_glrlm_RunVariance (indicating fibrosis and neovascularization probably). Oikonomou et al reported that CT radiomic texture features in coronary PVAT were more effective than mean attenuation in detecting histological changes such as fibrosis and neovascularization [35]. Similarly, in patients with myocardial infarction, radiomic texture features outperformed mean attenuation in characterizing pericoronary adipose tissue [36].

This study has several limitations. First, as a single-center study, it lacks external validation from other institutions. However, the independent equipment testing and prospective design partially address this concern. Second, the sample size is limited due to the dual requirement of specimen collection and CTA imaging. Although we utilized SVM—an algorithm well-suited for small datasets—to reduce overfitting, this limitation persists. Third, this study lacks an investigation into pathological alterations in PVAT and its specimen examination. Furthermore, as radiomic features are derived from predefined handcrafted features, the depth of feature exploration is inherently limited, and their biological basis has not been proven.

In conclusion, this study proposed a novel CTA-based carotid PVAT radiomics model to identify plaque vulnerability at the histological level, offering a non-invasive and convenient approach with strong diagnostic efficacy, interpretability, and generalizability. Future research on PVAT radiomics holds the potential to develop innovative non-invasive imaging biomarkers, thereby enhancing surgical decision-making and prognostic assessment for patients with CAS.

## Supplementary information

Supplementary information

## Data Availability

The raw data supporting the conclusions of this article will be made available by the authors. Requests to access these datasets should be directed to K.S.

## Abbreviations

AUC: Area under the curve
CAS: Carotid artery stenosis
CEA: Carotid endarterectomy
COAS-CAS: The Cohort of Optimized Assessment Strategy of Carotid Artery Stenosis
CTA: Computed tomography angiography
DCA: Decision curve analysis
ICC: Intraclass correlation coefficient
LASSO: Least absolute shrinkage and selection operator
LR: Logistic regression
ML: Machine learning
NPV: Negative predictive value
PPV: Positive predictive value
PUMCH: Peking Union Medical College Hospital
PVAT: Perivascular adipose tissue
Rad-score: Radiomic score
RF: Random forest
ROC: Receiver operating characteristic
ROI: Region of interest
SHAP: Shapley additive explanations
SVM: Support vector machine

## Declarations

### Study design

A prospective study.
