# Supplementary material for: Identification of histological carotid plaque vulnerability by CT angiography using perivascular adipose tissue radiomics signature
Source: Insights Imaging. 2026 Jan 5;17:2. doi: 10.1186/s13244-025-02134-y (PMC12770126; doi:10.1186/s13244-025-02134-y)

Identification of Histological Carotid Plaque Vulnerability by CT Angiography Using Perivascular Adipose Tissue Radiomics Signature

ELECTRONIC SUPPLEMENTARY MATERIAL

METHODS

Surgical specimen and histological analysis

Carotid plaque specimens were immediately obtained after surgical resection. Intraoperatively, complete excision was ensured, and the specimens were gently rinsed with saline to remove blood and other impurities. Then, they were immediately fixed with 10% formalin and decalcified with 10% acetic acid. Plaque segments with the highest stenosis area and plaque burden were sectioned at 4 micrometers, stained with hematoxylin-eosin, Masson and labeled with CD68 antibody to detect histological structure and inflammation. The plaques were characterized by two vascular pathologists using the American Heart Association classification.[1; 2] Plaques were classified as vulnerable according to the following patho-histologic features: active inflammation; thin cap with large lipid core; endothelial denudation with superficial platelet aggregation; fissured plaque; intraplaque hemorrhage; stenosis > 90%.[3] The pathologists were blinded to all demographic, clinical, and imaging information of the patients. Disagreements (occurred <5% of the time) were resolved by consensus.

Assessment of conventional plaque features in CT

To calculate the percentage of CAS, two methods were utilized: the European Carotid Surgery Trial (ECST) method and the North American Symptomatic Carotid Endarterectomy Trial (NASCET) method. On CTA, three measurements were taken: the luminal diameter at the distal normal segment of the carotid artery (a), the simulated luminal diameter at the carotid bulb (c), and the width of the narrowest segment of the internal carotid artery (b). The percentage of diameter stenosis was calculated using the following formulas: NASCET degree = (1-b/a) × 100%, ECST = (1-b/c) × 100%. Near occlusions were excluded from percentage stenosis calculation and were defined as 95%.[4]

Plaque thickness, including total plaque, soft plaque and calcified plaque thickness, was measured in the transverse axis with the highest stenosis area. Calcified plaque was defined as the region with an average attenuation of 130 HU or greater that occupied more than 50% of the total plaque volume.[5] The rim sign was characterized by adventitial calcification measuring ≤2 mm in thickness with adjacent soft plaque measuring ≥2 mm in thickness.[5] Spotty calcification was defined as a high-density component greater than 130 HU and less than 3 mm in size, surrounded by non-calcified plaque tissue.[6] Plaque ulceration was defined as an extension of contrast material of >1 mm into the atherosclerotic plaque on at least 2 orthogonal slices.[7; 8] Intraluminal thrombus was identified as an intraluminal filling defect on CTA.[9] Plaque burden was calculated as (1- lumen area/ vessel area at maximal stenosis)×100%.[10] Plaque length was defined as the maximum longitudinal length of the plaque. Remodeling index was calculated as (vessel area at maximal stenosis/ the vessel area of the distal vessel unaffected by plaque)×100%.[10] Metrics such as total plaque density (HU), total plaque volume (mm^3), calcified plaque volume (mm^3), and calcification proportion were calculated based on the plaque region of interest (ROI) manually segmented by doctors in 3D Slicer (version 5.0.3). All measurements were conducted using the PACS system and 3D Slicer (version 5.0.3) following standardized protocols.

The formula for calculating the Rad-score

*Rad-score = 0.4482758620689654 - 0.131270 * exponential_gldm_DependenceEntropy*

*+ 0.040919 * exponential_glszm_SmallAreaLowGrayLevelEmphasis*

*+ 0.063687 * lbp_3D_m1_glszm_GrayLevelNonUniformityNormalized*

*- 0.012630 * log_sigma_2_0_mm_3D_glcm_InverseVariance*

*+ 0.004784 * log_sigma_2_0_mm_3D_glrlm_RunVariance*

*- 0.012863 * logarithm_firstorder_RootMeanSquared*

*+ 0.008668 * original_glszm_SmallAreaLowGrayLevelEmphasis*

*-0.005286 * square_gldm_DependenceNonUniformityNormalized*

*+ 0.088327 * wavelet_HHL_firstorder_Skewness*

*-0.026117 * wavelet_LHL_firstorder_Skewness*

*+ 0.057920 * wavelet_LLL_glcm_InverseVariance*

*+ 0.037369 * wavelet_LLL_gldm_LargeDependenceLowGrayLevelEmphasis*

Reference

1 Stary HC, Chandler AB, Dinsmore RE et al (1995) A definition of advanced types of atherosclerotic lesions and a histological classification of atherosclerosis. A report from the Committee on Vascular Lesions of the Council on Arteriosclerosis, American Heart Association. Circulation 92:1355-1374

2 Stary HC (2000) Natural history and histological classification of atherosclerotic lesions: an update. Arterioscler Thromb Vasc Biol 20:1177-1178

3 Naghavi M, Libby P, Falk E et al (2003) From vulnerable plaque to vulnerable patient: a call for new definitions and risk assessment strategies: Part I. Circulation 108:1664-1672

4 Fox AJ (1993) How to measure carotid stenosis. Radiology 186:316-318

5 Saba L, Loewe C, Weikert T et al (2023) State-of-the-art CT and MR imaging and assessment of atherosclerotic carotid artery disease: the reporting-a consensus document by the European Society of Cardiovascular Radiology (ESCR). Eur Radiol 33:1088-1101

6 Motoyama S, Ito H, Sarai M et al (2015) Plaque Characterization by Coronary Computed Tomography Angiography and the Likelihood of Acute Coronary Events in Mid-Term Follow-Up. J Am Coll Cardiol 66:337-346

7 Lovett JK, Gallagher PJ, Hands LJ, Walton J, Rothwell PM (2004) Histological correlates of carotid plaque surface morphology on lumen contrast imaging. Circulation 110:2190-2197

8 de Weert TT, Cretier S, Groen HC et al (2009) Atherosclerotic plaque surface morphology in the carotid bifurcation assessed with multidetector computed tomography angiography. Stroke 40:1334-1340

9 Menon BK, Singh J, Al-Khataami A, Demchuk AM, Goyal M (2010) The donut sign on CT angiography: an indicator of reversible intraluminal carotid thrombus? Neuroradiology 52:1055-1056

10 Zhang R, Zhang Q, Ji A, Lv P, Zhang J, Fu C, Lin J (2021) Identification of high-risk carotid plaque with MRI-based radiomics and machine learning. Eur Radiol 31:3116-3126

Table S1. Clinical and imaging characteristics among the training, validation set and test set

Variables	Total (n = 122)	Train (n = 58)	Internal validation (n = 25)	Equipment independent test (n = 39)	*P*
Age	66.45 ± 7.74	65.48 ± 8.74	68.28 ± 6.73	66.72 ± 6.62	0.31
BMI	24.73 ± 2.69	24.66 ± 2.45	24.06 ± 3.20	25.25 ± 2.64	0.22
Sex					0.46
Male	100 (81.97)	45 (77.59)	22 (88.00)	33 (84.62)	
Female	22 (18.03)	13 (22.41)	3 (12.00)	6 (15.38)	
Hypertension	84 (68.85)	41 (70.69)	18 (72.00)	25 (64.10)	0.74
Diabetes,	44 (36.07)	18 (31.03)	13 (52.00)	13 (33.33)	0.17
Hyperlipemia	48 (39.34)	26 (44.83)	7 (28.00)	15 (38.46)	0.35
CAD	32 (26.23)	11 (18.97)	7 (28.00)	14 (35.90)	0.17
MI	6 (4.92)	1 (1.72)	1 (4.00)	4 (10.26)	0.15
Smoking	79 (64.75)	36 (62.07)	15 (60.00)	28 (71.79)	0.53
TC	3.62 ± 0.84	3.59 ± 0.85	3.44 ± 0.79	3.78 ± 0.84	0.26
HDL	0.99 ± 0.23	1.00 ± 0.23	0.97 ± 0.26	1.00 ± 0.19	0.81
LD	2.00 ± 0.67	2.00 ± 0.73	1.77 ± 0.50	2.16 ± 0.65	0.08
TG	1.09 (0.89, 1.73)	1.09 (0.83,1.72)	1.02 (0.68,1.66)	1.15 (0.95,1.70)	0.41
Symptom					0.26
Asymptomatic	84 (68.85)	42 (72.41)	19 (76.00)	23 (58.97)	
Symptomatic	38 (31.15)	16 (27.59)	6 (24.00)	16 (41.03)	
NASCET category					0.28
50%-69%	47 (38.52)	20 (34.48)	8 (32.00)	19 (48.72)	
70%-99%	75 (61.48)	38 (65.52)	17 (68.00)	20 (51.28)	
NASCET degree	0.75 (0.64, 0.80)	0.75 (0.69,0.82)	0.75 (0.66,0.78)	0.71 (0.62,0.78)	0.06
ECST degree	0.79 ± 0.09	0.80 ± 0.10	0.78 ± 0.11	0.78 ± 0.07	0.41
Stability					0.16
Vulnerable	77 (63.11)	32 (55.17)	16 (64.00)	29 (74.36)	
Stable	45 (36.89)	26 (44.83)	9 (36.00)	10 (25.64)	

Total plaque thickness	4.87 ± 1.44	4.88 ± 1.53	4.70 ± 1.44	4.95 ± 1.32	0.78
Soft plaque thickness	3.86 ± 1.60	3.89 ± 1.43	3.43 ± 2.06	4.11 ± 1.50	0.24
Calcified plaque thickness	1.82 (1.37, 2.21)	1.73 (1.05,2.19)	1.81 (1.61,2.79)	1.93 (1.66,2.21)	0.24
Calcified plaque	34 (27.87)	13 (22.41)	11 (44.00)	10 (25.64)	0.12
Rim Sign	38 (31.15)	20 (34.48)	6 (24.00)	12 (30.77)	0.64
Spotty calcification,	24 (19.67)	11 (18.97)	6 (24.00)	7 (17.95)	0.82
Plaque ulceration	16 (13.11)	7 (12.07)	5 (20.00)	4 (10.26)	0.50
Intraluminal thrombus	6 (4.92)	4 (6.90)	1 (4.00)	1 (2.56)	0.86
Total plaque density	168.13 (100.56, 258.50)	164.70 (96.01,249.05)	166.42 (113.25,415.67)	193.46 (116.32,241.49)	0.61
Total plaque volume	670.67 (341.80, 945.14)	705.87 (431.90,908.43)	615.00 (287.30,1216.57)	623.35 (292.27,924.75)	0.69
Calcified plaque volume	185.19 (69.45, 389.38)	235.96 (65.37,430.40)	180.37 (70.13,363.31)	169.90 (72.67,351.50)	0.96
Calcification proportion	0.34 (0.18, 0.55)	0.33 (0.17,0.48)	0.36 (0.17,0.71)	0.34 (0.22,0.49)	0.73
Plaque burden	0.89 (0.82, 0.92)	0.89 (0.83,0.94)	0.89 (0.82,0.91)	0.89 (0.81,0.92)	0.57
Plaque length	19.26 (13.63, 23.18)	19.11 (14.71,22.14)	20.46 (14.28,24.10)	18.29 (12.55,24.61)	0.59
Remodeling index	2.24 ± 1.04	2.33 ± 1.16	2.20 ± 0.71	2.12 ± 1.04	0.61
PVAT attenuation	-70.72 ± 7.56	-71.04 ± 7.37	-70.24 ± 7.71	-70.55 ± 7.92	0.89

Categorical variables are presented as number (%). Continuous variables are presented as mean ± standard deviation or median (interquartile range). BMI, Body mass index; CAD, coronary heart disease; MI, myocardial infarction; TC, Total Cholesterol; HDL, High-Density Lipoprotein; LDL, Low-Density Lipoprotein; TG, Triglycerides; NASCET, the North American Symptomatic Carotid Endarterectomy Trial; ECST, the European Carotid Surgery Trial; PVAT, perivascular adipose tissue.

Table S2. Conventional plaque features and PVAT attenuation of vulnerable and stable plaque in all patients

Variables	Total (n = 122)	Vulnerable (n = 77)	Stable (n = 45)	*P*
**Conventional plaque features**				
Total plaque thickness	4.87 ± 1.44	5.06 ± 1.41	4.54 ± 1.45	0.06
Soft plaque thickness	3.86 ± 1.60	4.09 ± 1.62	3.49 ± 1.51	0.046*
Calcified plaque thickness	1.82 (1.37, 2.21)	1.83 (1.45, 2.33)	1.80 (1.25, 2.19)	0.43
Calcified plaque	34 (27.87)	21 (27.27)	13 (28.89)	0.85
Rim Sign	38 (31.15)	23 (29.87)	15 (33.33)	0.69
Spotty calcification	24 (19.67)	15 (19.48)	9 (20.00)	0.94
Intraluminal thrombus	6 (4.92)	2 (2.60)	4 (8.89)	0.26
Plaque ulceration	16 (13.11)	14 (18.18)	2 (4.44)	0.03*
Total plaque density	168.13 (100.56, 258.50)	177.95 (95.45, 257.41)	164.89 (119.10, 265.36)	0.76
Total plaque volume	670.67 (341.80, 945.14)	692.37 (443.67, 967.84)	431.83 (256.59, 844.74)	0.03*
Calcified plaque volume	185.19 (69.45, 389.38)	199.87 (74.10, 461.18)	180.28 (59.44, 365.04)	0.33
Calcification proportion	0.34 (0.18, 0.55)	0.31 (0.17, 0.51)	0.36 (0.22, 0.59)	0.36
Plaque burden	0.89 (0.82, 0.92)	0.89 (0.82, 0.92)	0.86 (0.81, 0.92)	0.37
Plaque length	19.26 (13.63, 23.18)	19.48 (14.65, 23.52)	18.58 (12.63, 21.86)	0.33
Remodeling index	2.24 ± 1.04	2.21 ± 0.83	2.28 ± 1.33	0.77
**PVAT feature**				
PVAT attenuation	-70.72 ± 7.56	-69.27 ± 6.74	-73.20 ± 8.29	0.005*

PVAT, perivascular adipose tissue; OR, Odds Ratio; CI, Confidence Interval.

Categorical variables are presented as number (%). Continuous variables are presented as mean ± standard deviation or median (interquartile range).

*p < 0.05, indicating that the difference was statistically significant

Table S3. Plaque characteristics among patients scanning by four different equipment

Variables	Total (n = 122)	Equipment A (n = 83)	Equipment B (n = 8)	Equipment C (n = 6)	Equipment D (n = 25)	P
**Total plaque thickness**	4.87 ± 1.44	4.83 ± 1.50	4.80 ± 1.36	4.40 ± 0.74	5.13 ± 1.41	0.671
**Soft plaque thickness**	3.86 ± 1.60	3.75 ± 1.64	4.06 ± 1.38	3.31 ± 0.78	4.32 ± 1.63	0.344
**Calcified plaque thickness**	1.82 (1.37, 2.21)	1.80 (1.17,2.24)	2.19 (1.97,2.37)	1.68 (1.58,1.88)	1.87 (1.47,2.41)	0.158
**Calcified plaque**	34 (27.87)	24 (28.92)	3 (37.50)	3 (50.00)	4 (16.00)	0.277
**Rim Sign**	38 (31.15)	26 (31.33)	3 (37.50)	2 (33.33)	7 (28.00)	0.945
**Spotty calcification**	24 (19.67)	17 (20.48)	2 (25.00)	1 (16.67)	4 (16.00)	0.889
**Plaque ulceration**	16 (13.11)	12 (14.46)	2 (25.00)	0 (0.00)	2 (8.00)	0.493
**Intraluminal thrombus**	6 (4.92)	5 (6.02)	1 (12.50)	0 (0.00)	0 (0.00)	0.432
**Total plaque density**	168.13 (100.56, 258.50)	164.89 (96.61,266.74)	252.97 (203.65,447.69)	204.81 (186.72,263.51)	153.88 (83.38,205.89)	0.055
**Total plaque volume**	670.67 (341.80, 945.14)	681.51 (398.44,970.93)	798.63 (423.32,964.50)	250.55 (246.99,647.70)	623.35 (302.88,906.94)	0.282
**Calcified plaque volume**	185.19 (69.45, 389.38)	199.87 (65.66,418.23)	388.55 (181.21,584.30)	128.61 (79.40,290.44)	92.51 (64.56,320.31)	0.234
**Calcification proportion**	0.34 (0.18, 0.55)	0.33 (0.17,0.57)	0.36 (0.33,0.69)	0.50 (0.34,0.64)	0.27 (0.16,0.40)	0.252
**Plaque burden**	0.89 (0.82, 0.92)	0.89 (0.82,0.93)	0.87 (0.82,0.90)	0.83 (0.74,0.89)	0.89 (0.84,0.92)	0.75
**Plaque length**	19.26 (13.63, 23.18)	19.28 (14.48,22.99)	22.36 (14.98,37.27)	16.20 (10.00,20.64)	16.90 (12.09,22.06)	0.425
**Remodeling index**	2.24 ± 1.04	2.29 ± 1.04	1.87 ± 0.89	1.95 ± 1.10	2.25 ± 1.08	0.635

Categorical variables are presented as number (%). Continuous variables are presented as mean ± standard deviation or median (interquartile range). PVAT, perivascular adipose tissue;

Figure S1. Number and proportion of extracted radiomics features. GLCM, Gray-Level Co-Occurrence Matrix; GLDM, Gray-Level Dependence Matrix; GLRLM, Gray-Level Run Length Matrix; GLSZM, Gray-Level Size Zone Matrix; NGTDM, Neighborhood Gray-Tone Difference Matrix.

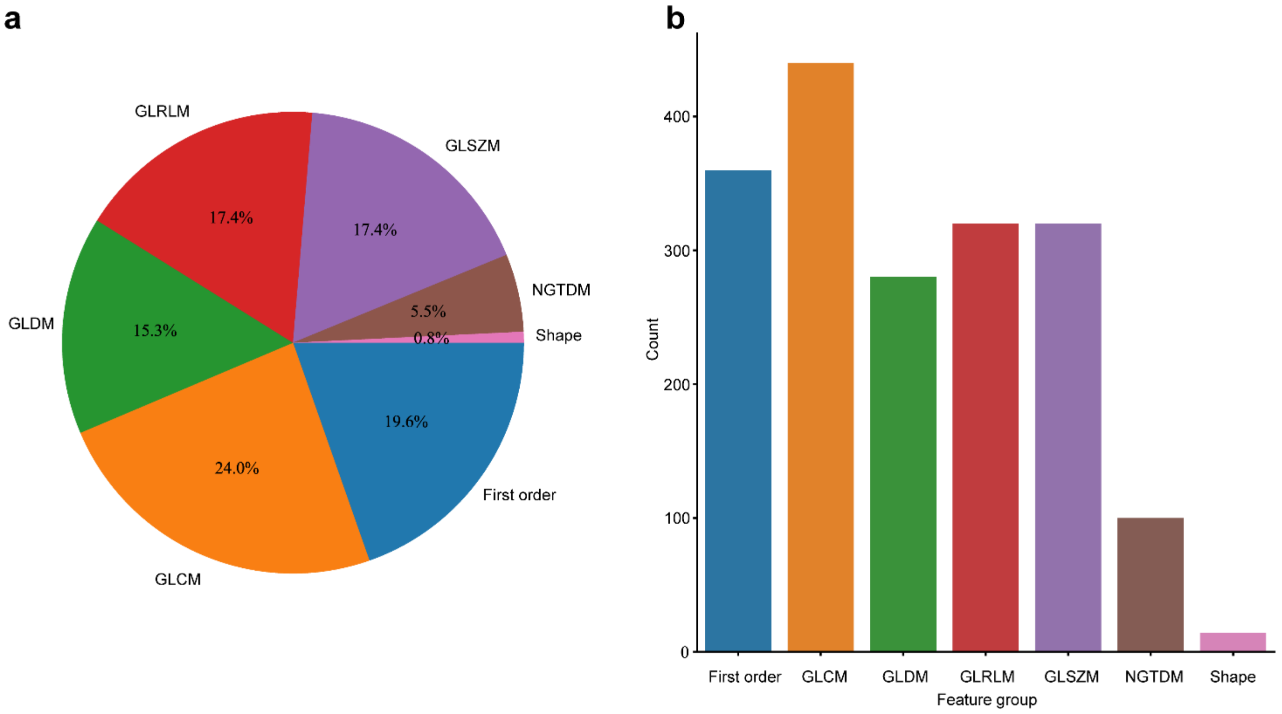

Figure S2. Violin plot of the statistical significance of seven types of radiomics features. GLCM, Gray-Level Co-Occurrence Matrix; GLDM, Gray-Level Dependence Matrix; GLRLM, Gray-Level Run Length Matrix; GLSZM, Gray-Level Size Zone Matrix; NGTDM, Neighborhood Gray-Tone Difference Matrix.

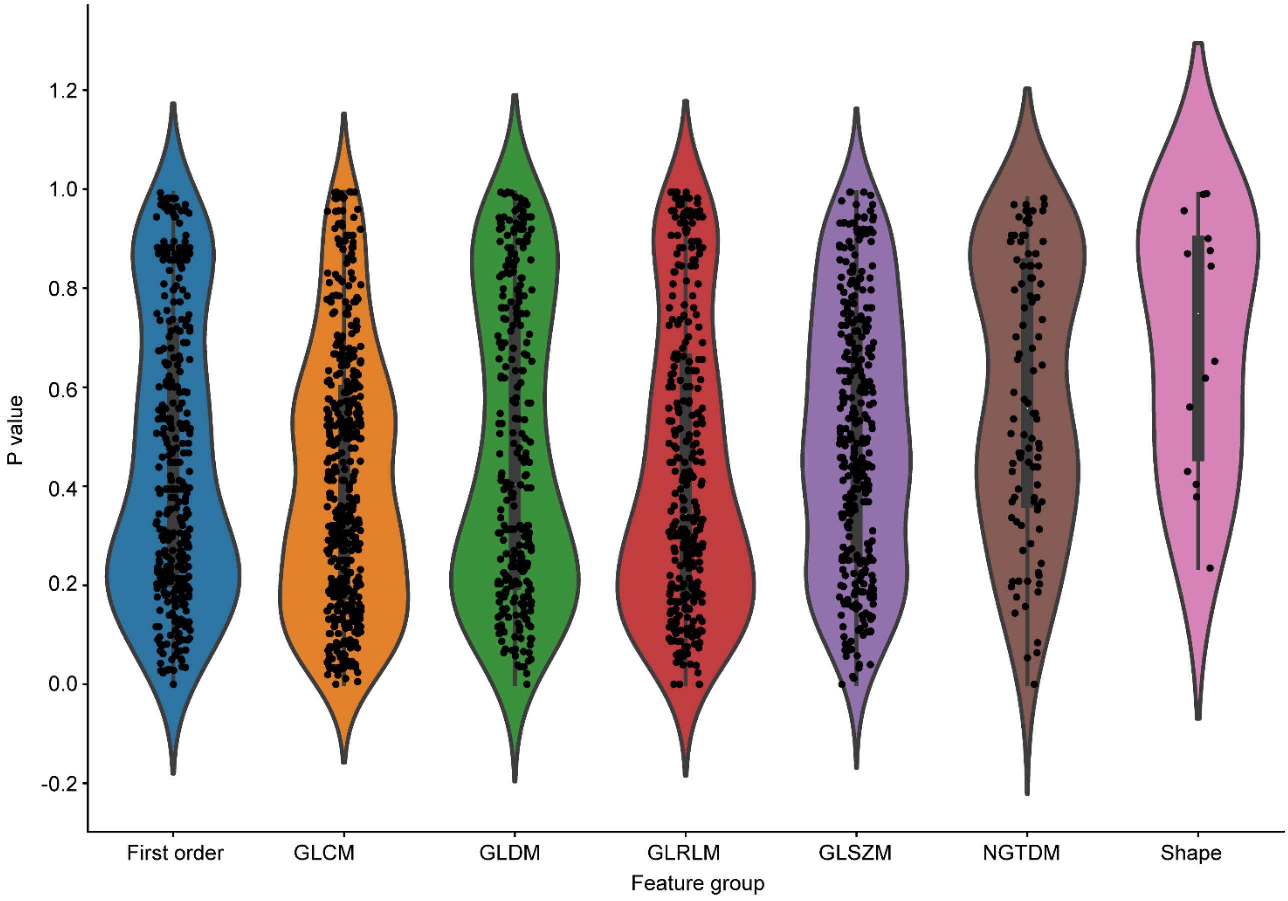

Figure S3. Heatmap of spearman correlation coefficients for 64 radiomics features.

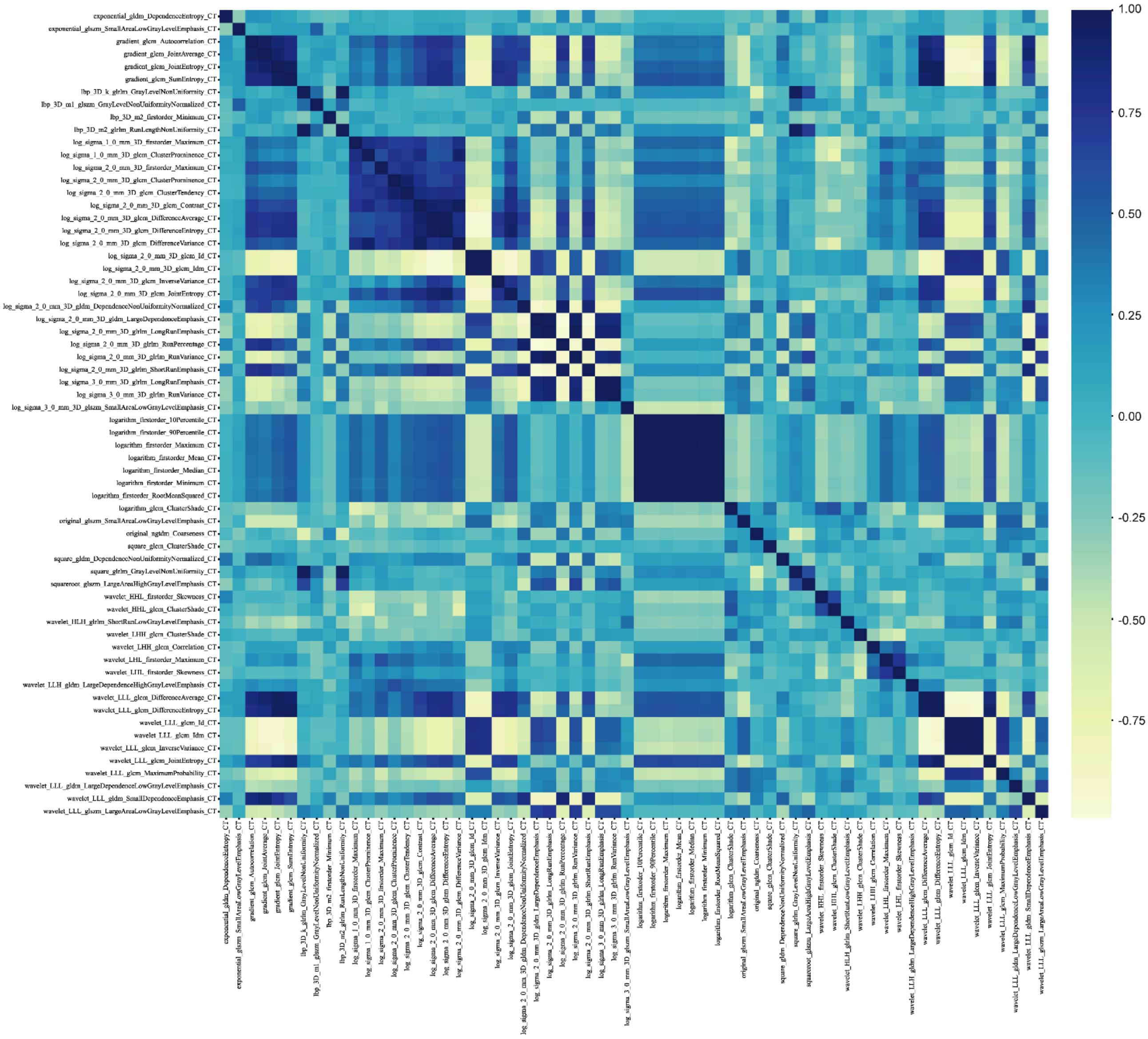

Figure S4. Radiomics feature selection by LASSO. The coefficients for feature selection in LASSO regression (**a**). The MSE of LASSO regression (**b**). The corresponding coefficients of selected radiomics features (**c**). LASSO, least absolute shrinkage and selection operator. MSE, mean standard error.

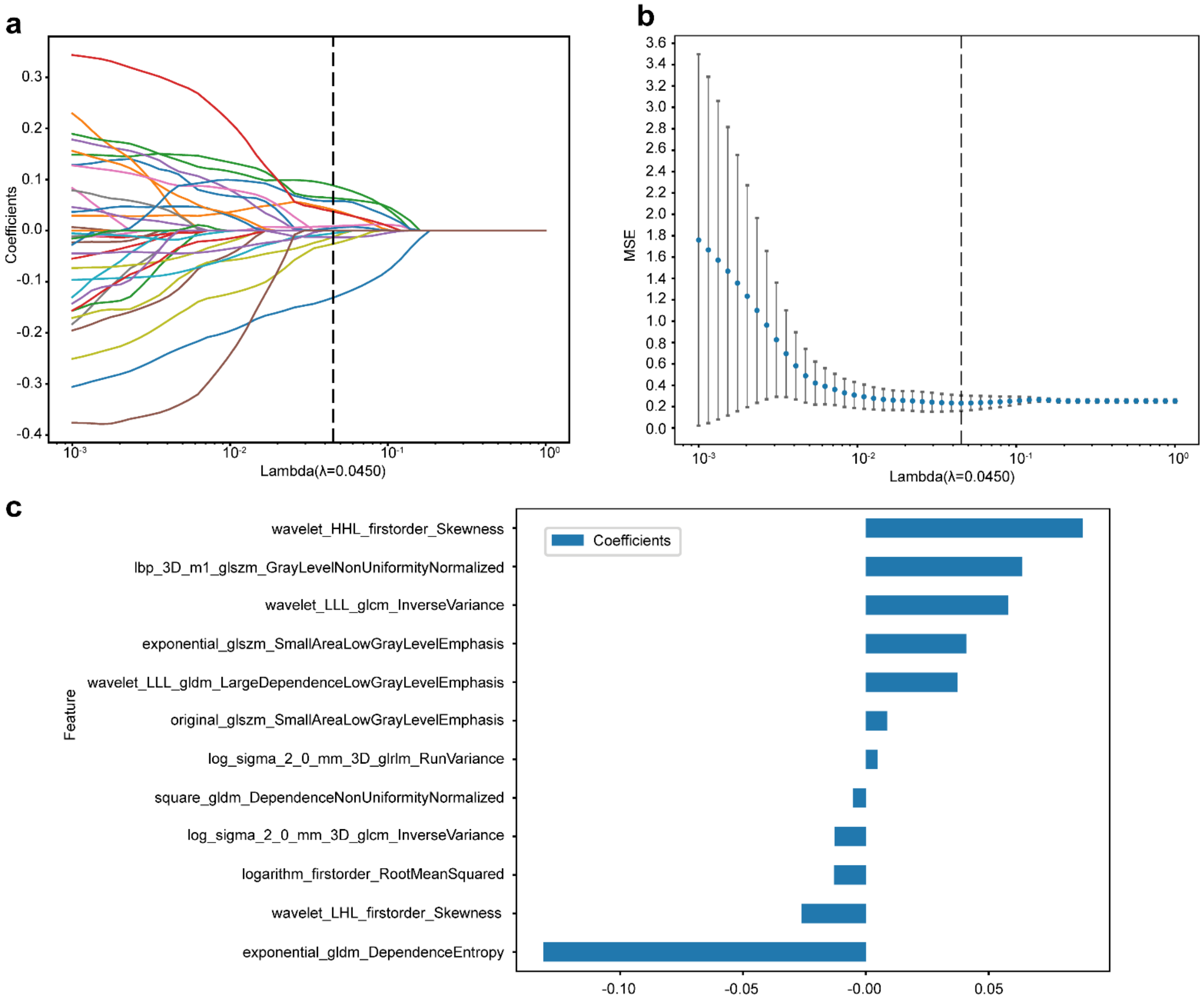

Figure S5. Receiver operating characteristic (ROC) curves of three ML models on the training set (a), internal validation set (b) and independent equipment test set (c). SVM, Support Vector Machine; LR, Logistic Regression; RF, Random Forest.

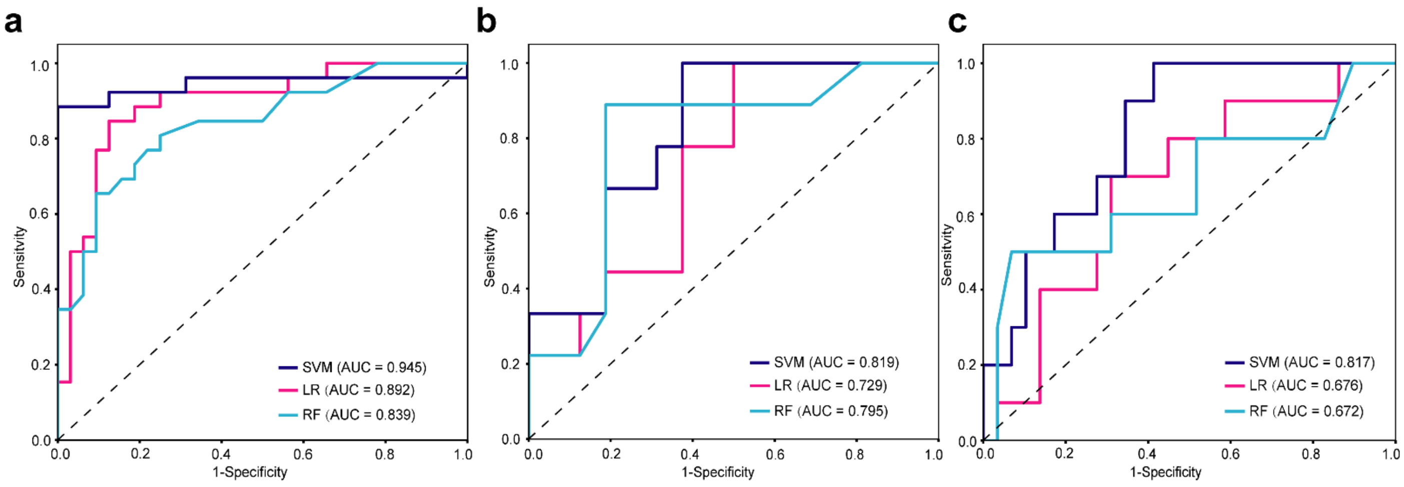

Supplement: Supplementary file 1 — Supplementary information [file 13244_2025_2134_MOESM1_ESM.pdf]
